# Scar quantification by cardiovascular magnetic resonance as an independent predictor of long-term survival in patients with ischemic heart failure treated by coronary artery bypass graft surgery

**DOI:** 10.1186/s12968-016-0265-y

**Published:** 2016-07-18

**Authors:** Krishna Kancharla, Gaby Weissman, Abdalla A. Elagha, Kalyan Kancherla, Swetha Samineni, Peter C. Hill, Steven Boyce, Anthon R. Fuisz

**Affiliations:** Department of cardiology, Mayo Clinic, Rochester, 55905 MN USA; Division of Cardiology, MedStar Heart and Vascular Institute, MedStar Washington Hospital Center, Washington, DC USA; Georgetown University, Washington, DC USA; Department of cardiology, Cairo University Hospitals, Cairo, Egypt; Translational Medicine Branch, National Heart, Lung, and Blood Institute, Bethesda, MD USA; MedStar Health Research Institute, Washington, DC USA; Howard University Hospital, Washington, DC USA; Mayo Clinic Health Systems, Austin, MN USA; Department of Cardiac Surgery, MedStar Heart and Vascular Institute, MedStar Washington Hospital Center, Washington, DC USA; Division of Cardiology, Westchester Medical Center, Valhalla, New York, USA

**Keywords:** Cardiovascular magnetic resonance, Scar, Ischemic cardiomyopathy, CABG, Mortality

## Abstract

**Background:**

Scar burden by late gadolinium enhancement (LGE) cardiovascular magnetic resonance (CMR) is associated with functional recovery after coronary artery bypass surgery (CABG). There is limited data on long-term mortality after CABG based on left ventricular (LV) scar burden.

**Methods:**

Patients who underwent LGE CMR between January 2003 and February 2010 within 1 month prior to CABG were included. A standard 16 segment model was used for scar quantification. A score of 1 for no scar, 2 for ≤ 50 % and 3 for > 50 % transmurality was assigned for each segment. LV scar score (LVSS) defined as the sum of segment scores divided by 16. All-cause mortality was ascertained by social security death index.

**Results:**

One hundred ninety-six patients met the inclusion criteria. 185 CMR studies were available. History of prior MI was present in 64 % and prior CABG in 5.4 % of patients. Scar was present in 72 % of patients and median LVEF was 38 %. Over a median follow up of 8.3 years, there were 64 deaths (34.6 %). There was no statistically significant difference in mortality between Scar and No-scar groups (37 % versus 29 %). In the group with scar, a lower scar burden (defined either < 4 segments with scar or based on LVSS) was independently associated with increased survival.

**Conclusion:**

In patients undergoing surgical revascularization, scar burden is negatively associated with survival in patients with scar. However, there is no difference in survival based on presence or absence of scar alone. CMR prior to CABG adds additional prognostic information.

## Background

Coronary artery bypass graft surgery (CABG) is one of the most common cardiac surgeries performed worldwide. The decision for surgical revascularization is based on several factors including patient characteristics, coronary anatomy and presence of ischemia or infarct. Cardiovascular Magnetic Resonance (CMR) is considered gold standard for assessment of fibrosis/scar and is commonly used in evaluation of patients with significant coronary artery disease (CAD) and cardiomyopathy prior to formulating treatment strategy.

Assessment of size and extent of scar or fibrosis by Late Gadolinium Enhancement (LGE) by CMR is accurate and reproducible [1–5]. The presence and amount of LGE is an independent predictor of cardiovascular outcomes and mortality in patients with ischemic heart failure (HF) and non-ischemic cardiomyopathies (NICM) [6–8]. LGE is effective in detection and assessment of both acute and chronic myocardial infarction (MI) and is well matched to the perfusion territory of infarct related artery [9]. In patients with CAD, LGE is a predictor of major adverse cardiac events (MACE) and mortality beyond common clinical factors or coronary anatomy and is independent of left ventricular ejection fraction (LVEF) [10–13].

In ischemic HF, the transmurality of the scar is negatively associated with functional recovery. Segments with > 50 % scar have a low probability of functional recovery [14–16]. The presence of ≥ 10 segments without scar or with <50 % transmural scar predicted a global functional recovery in one study [17] while the presence of ≤4 scar segments predicted global functional recovery after CABG in another study [18]. In patient with ischemic HF observational studies support improved survival with CABG over medical therapy during 10 year follow up [19, 20] while a more recent randomized study showed a reduction in cardiac mortality but no difference in all-cause mortality [21, 22]. In a cohort of patients treated with CABG, the presence of larger end systolic volume index and scar burden on CMR was noted to derive survival benefit compared to treatment with medical therapy alone [23].

While there is data to support functional improvement post revascularization, there is limited data in the use of CMR in outcomes after revascularization. In this study, we evaluated the association between LGE burden and long-term mortality after CABG.

## Methods

Patients who underwent CMR with LGE between January 2003 and February 2010 within 1 month prior to CABG surgery were included.

A comprehensive surgical database maintained at our institution was accessed to obtain demographic, clinical and surgical information. Angiographic data including major and branch vessel stenosis was collected. All-cause mortality was ascertained by social security death index search.

### CMR protocol and analysis

Patients were positioned supine in a clinical 1.5 T MR scanner (CV Intera, Philips, Best, The Netherlands). All images were acquired using a cardiac phased-array receiver coil during breath-holds (≈8 s), together with respiratory and cardiac gating. Cine images were acquired through the entire left ventricle using 6-mm-thick slices to minimize the effects of partial volume. LGE images were obtained after intra venous contrast agent administration (0.2 mmol/kg gadolinium dimeglumine, until July 2006, then 0.1 mmol/kg gadobenate dimegluine) [2] using a segmented inversion-recovery sequence, with an in-plane spatial resolution of 1.3 to 1.7 mm [1].

**Fig. 1:**
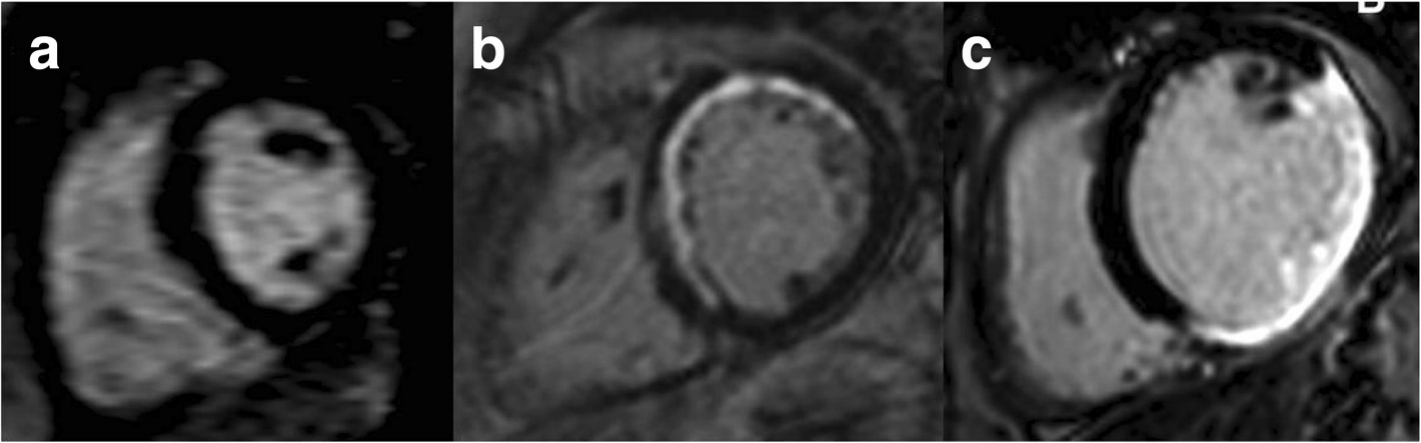
LGE on CMR in 3 different patients. **a**- No scar, **b**- Less than 50 % thickness involvement in multiple segments and **c**- More than 50 % thickness involvement in multiple segments

Evaluation of CMR and post-processing were performed on a commercially available workstation (View Forum, Philips Medical Systems). End-diastolic and end-systolic cardiac contours were traced manually on the series of LV short-axis cine slices to obtain LVEF and LV volumes. Scar quantification was performed by image review by an experienced observer who was blinded from clinical analysis and prior CMR reading. Based on the prior data on functional improvement after revascularization in patients with LGE by CMR, a simplified quantification method was used. Left ventricular (LV) was divided into a standard 16 segment model (6 basal, 6 mid and 4 apical segments) for quantification of scar. The number of segments with scar was recorded. Each segment was then scored based on scar transmurality. Segment was assigned a score of 1 for no scar, 2 for ≤50 % thickness, and 3 for > 50 % thickness or micro vascular obstruction (MVO) (Fig. 1). Subjects were divided into <4 segments of scar (group A) and ≥4 segments of scar (group B). LV scar score (LVSS) was calculated by the sum of the scores of all segments divided by 16 [10] (a LVSS of 1 would represent no scar).

### Statistical analysis

Data analysis was performed using SAS 9.3 Institute Inc., Cary, NC, USA. Categorical variables were presented as proportions and analyzed with Chi square test (Fisher’s exact test was used in case of small numbers). For comparison between continuous variables two sample t- test was used. Non-parametric Wilcoxon rank sum test was used in case of non-normal variables. Univariate and multivariate analysis was performed with Cox proportional hazard model. Multivariate analysis was performed using variables that have significant association in the bivariate analysis. Survival probability was plotted with Kaplan Meier curves (Log rank test). A value of *p* < 0.05 was considered statistically significant.

## Results

One hundred ninety-six patients met the inclusion criteria. 185 CMR studies were available. The median time from CMR study to CABG surgery was 2 (1, 4) days. The mean age of the study population at the time of surgery was 63.2 years (±11.5). Seventy-two percent were male and 66 % were Caucasians. History of prior MI was present in 64 % of patients and prior CABG in 5.4 % patients. All patients in the study group had significant stenosis (≥70 %) in at least one major epicardial vessel. There was a median of 2 (1,3) significantly stenosed vessels and 1 (0,1) total occlusions. All patients were deemed appropriate for CABG as the revascularization strategy by the angiographer and patient’s cardiologist due to the presence of complex coronary artery disease. A median of 4 (3, 4) vessels (major or branch vessels) were bypassed. Scar was present in 72 % of patients and median LVEF was 38 % (28, 52). Over a median follow up of 8.3 years (7, 10), there were 64 deaths (34.6 %) in the entire group.

Patients were categorized into Scar group and No-scar groups. Scar group was further categorized into two groups based on the number of segments with scar (<4 segments versus ≥4 segments with scar) and also into three groups based on LV scar score (LVSS 1–1.24, LVSS 1.25–1.42 and LVSS >1.42).

### Scar versus No-scar group

**Fig. 2:**
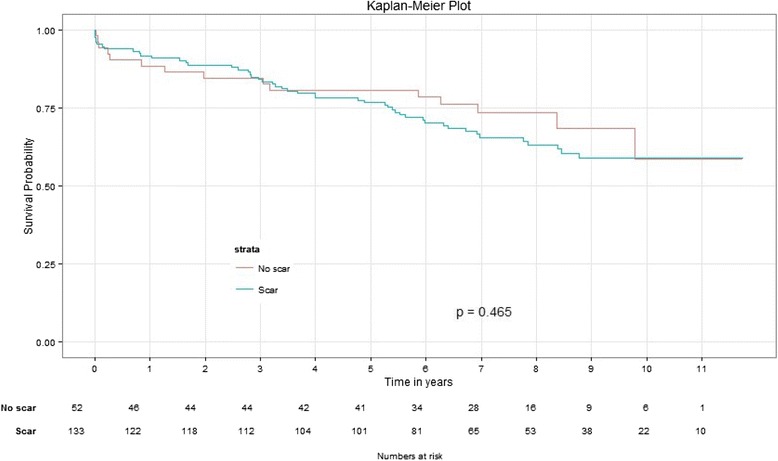
Survival probabilities between No scar and Scar group

There were 133 patients in the scar group and 52 patients in the No-scar group. The baseline characteristics are shown in Table 1. Patients in Scar group had statistically higher proportion of men (78 % vs. 56 % (*p* = 0.002)), history of prior MI (74 % vs. 38 % (*p* < 0.0001)) and lower percent LVEF (39 % versus 45 % (*p* = 0.038)) compared to patients in the No-scar group. During the follow up there was no statistically significant difference (Fig. 2) in long-term mortality between Scar and No-scar groups (37 % versus 29 %, *p* = 0.47)

**Table 1:** Baseline characteristics of patients at the time of CABG

Parameter	All patients Mean/Median (IQ)/%	No-scar Mean/Median (IQ)/%	Scar group Mean/Median (IQ)/%	*p* value
Number of patients	185	52	133	
Age (years)	63.2 (11.5)	64.2 (9.7)	62.8 (12.2)	0.47
Male (%)	71.9	55.8	78.2	0.002
Body Mass Index(BMI)	28.3 (5.1)	28.0 (5.8)	28.4 (4.8)	0.60
History Of Smoking (%)	63.2	57.7	65.4	0.33
Diabetes Mellitus (%)	34.6	25.0	38.3	0.09
Hypertension (%)	78.4	76.9	78.9	0.76
History of Prior MI (%)	63.8	38.5	73.7	<0.0001
Cerebrovascular Disease (%)	14	15.4	13.5	0.75
Peripheral artery disease (%)	20	23.1	18.8	0.5130
Dyslipidemia (%)	70.8	65.4	72.9	0.31
Previous CABG history (%)	5.4	0.0	7.5	0.06
Glomerular filtration rate	68.32 (25.20)	66.0 (25.7)	69.2 (25.0)	0.44
Chronic Lung Disease (%)	17.8	21.1	16.5	0.46
B - Blocker (%)	78.9	73.1	81.2	0.2232
ACE - Inhibitor (%)	56.8	51.9	58.7	0.4067
Aspirin (%)	91.4	86.5	93.2	0.1548
Lipid lowering therapy (%)	71.6	64.3	74.8	0.2076
LVEDV (ml)	210.75 (77.79)	195.44 (86.20)	216.82 (73.65)	0.0936
LVESV (ml)	134.29 (77.40)	118.88 (86.76)	140.40 (72.80)	0.0899
ESVi (ml/m2)	29.77 (18.53, 46.19)	24.67 (12.62, 41.66)	32.39 (19.84, 46.98)	0.0424
LVEF (%)	38 (28,52)	45 (25,65)	37 (28,49)	0.038
Number of Segments scar	2 (0,5)	0	3 (2,6)	NA
STS % mortality risk	1.4 (0.8,2.8)	1.4 (0.7,2.6)	1.4 (0.7,2.9)	0.71
Cardiopulmonary Bypass (%)	63.8	61.5	64.7	0.6911
Number of vessels bypassed	4 (3–4)	3.5 (2–4)	4 (3–4)	0.5929

*MI* Myocardial Infarction, *ACE* Inhibitor = Angiotensin convertase enzyme Inhibitor, *LVEDV* left ventricular end diastolic volume, *LVESV* left ventricular end systolic volume, *ESVi* end systolic volume index, *LVEF* left ventricular ejection fraction, *STS* society of thoracic surgery

### Scar group analysis

#### Less than 4 segments scar group (A) versus ≥ 4 segments scar group (B)

The baseline characteristics (Table 2) were similar except that patients in group B had a higher proportion of history of prior CABG (14 % vs. 1.5 % *p* = 0.009) and lower proportion of history of ACE inhibitor usage (47 % versus 70 % (*p* = 0.0066)) compared to patients in group A. There was no statistically significant difference in STS scores or LVEF between the two groups. The median number of segments of LGE was 2 (1, 3) for patients in group A vs. 6 (5, 7) in group B. The number of trans mural segments was 1(0,2) vs. 2(1,5), *p* < 0.0001) and the LVSS was 1.2(1.1,1.3) vs. 1.5(1.4,1.7) *p* < 0.0001) in group A vs. group B respectively. During the follow up 27 % patients died in group A vs. 47 % group B (*p* = 0.02).

**Table 2:** Baseline characteristics among Scar groups at the time of CABG

Parameter	<4 segments - Group A (Mean/Median (IQ)/%	≥4 segments - Group B Mean/Median (IQ)/%	*p* value
Number	67	66	
Age (Years)	63.3 (11.2)	62.3 (13.2)	0.69
Male (%)	76.1	80.3	0.56
Body Mass Index (BMI)	28.4 (4.7)	28.4 (4.9)	0.98
History Of Smoking (%)	62.7	68.2	0.51
Diabetes Mellitus (%)	38.8	37.9	0.91
Hypertension (%)	79.1	78.8	0.97
History of Prior MI (%)	74.6	72.7	0.81
Cerebrovascular Disease (%)	14.9	12.1	0.64
Peripheral artery disease (%)	14.9	22.7	0.25
Dyslipidemia (%)	71.6	74.2	0.74
Previous CABG (%)	1.5	13.6	0.009
Glomerular filtration rate (GFR)	68.9 (22.0)	69.6 (28.0)	0.87
Chronic Lung Disease (%)	19.4	13.6	0.37
B – Blocker (%)	85.1	77.3	0.2495
ACE - Inhibitor (%)	70.2	47.0	0.0066
Aspirin (%)	92.5	93.9	1.000
Lipid Lowering Therapy (%)	68.4	83.3	0.0914
LVEDV (ml)	206.8 (73.5)	226.7 (73.0)	0.1237
LVESV (ml)	129.4 (71.6)	151.3 (72.9)	0.0847
LVEF(%)	39 (28,52)	35 (26,45)	0.12
Number of Segments scar	2 (1,3)	6 (5,7)	<0.0001
Number of trans mural segments	1 (0, 2)	2 (1,5)	<0.0001
Scar score	1.19 (1.13,1.25)	1.50 (1.38,1.75)	<0.0001
STS % mortality risk	1.40 (0.66,3.59)	1.46 (0.79,2.69)	0.90
Cardiopulmonary Bypass Used (%)	62.7	66.7	0.6312
Number of vessels bypassed	4 (2–5)	3 (3–4)	0.5929

*MI* myocardial infarction, *ACE* angiotensin converting enzyme, *CABG* coronary artery bypass graft surgery, *LVEDV* left ventricular end diastolic volume, *LVESV* left ventricular end-systolic volume, *STS* society of thoracic surgeons, *LVEF* left ventricular ejection fraction

**Fig. 3:**
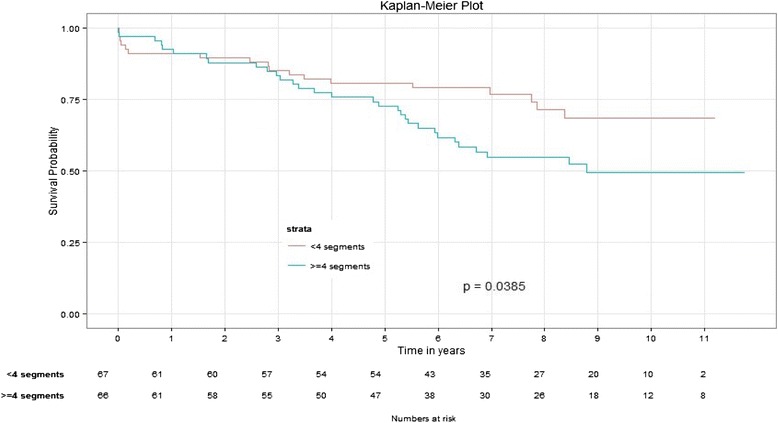
Survival probabilities between <4 segment scar and ≥ 4 segments scar groups

In a univariate analysis (Table 3) Age, LVEF, Female gender, presence of peripheral artery disease (PAD), GFR, and Scar group (A vs. B), number of segments of scar, number of transmural segments and LVSS were all significantly associated with long-term mortality. In multivariate analysis (Table 4) Scar group B was independently associated with long-term mortality (*p* = 0.018, HR = 2.0 (1.1–3.7). Age, LVEF and GFR were also independently associated with long-term mortality. Survival (Fig. 3) was significantly lower in group B compared to group A (*p* = 0.038)

**Table 3:** Scar group – Cox proportional hazard model, univariate analysis with long-term mortality

Parameter	*p* value	HR	CI
Age	<0.0001*	1.067	1.037–1.098
Female gender	0.0346*	1.927	0.049–3.541
Peripheral artery disease(PAD)	0.0319*	2.014	1.062–3.816
Glomerular filtration rate(GFR)	0.0003*	0.978	0.966–0.990
LVEF%	0.0079*	0.973	0.954–0.993
Number of segments	0.0040*	1.164	1.049–1.291
Segment group (≥4)	0.0416*	1.830	1.02–3.3
Scar score	0.0016*	4.435	1.756–11.202
Number of trans mural segments	0.0042*	1.174	1.052–1.310
STS mortality risk	<0.0001*	1.182	1.20–1.248

*MI* Myocardial infarction, *ACE* Angiotensin converting enzyme, *CABG* Coronary artery bypass graft surgery, *LVEDV* Left ventricular end diastolic volume, *LVESV* Left Ventricular End-Systolic Volume, *STS* society of thoracic surgeons. Asterisk represents variables with significant association

**Table 4:** Scar group – Cox proportional hazard model, multivariate analysis with long-term mortality

Parameter	*p* value	HR	CI
Segment group (≥4)	0.0187	2.035	1.126–3.681
Age	0.0002	1.061	1.029–1.095
LVEF	0.0063	0.969	0.948–0.991
Gender (Female)	0.1811	1.545	0.817–2.922
Peripheral artery disease(PAD)	0.2651	1.483	0.742–2.965
Glomerular filtration rate(GFR)	0.0132	0.989	0.967–0.996

### LV scar score and long-term mortality

**Fig. 4:**
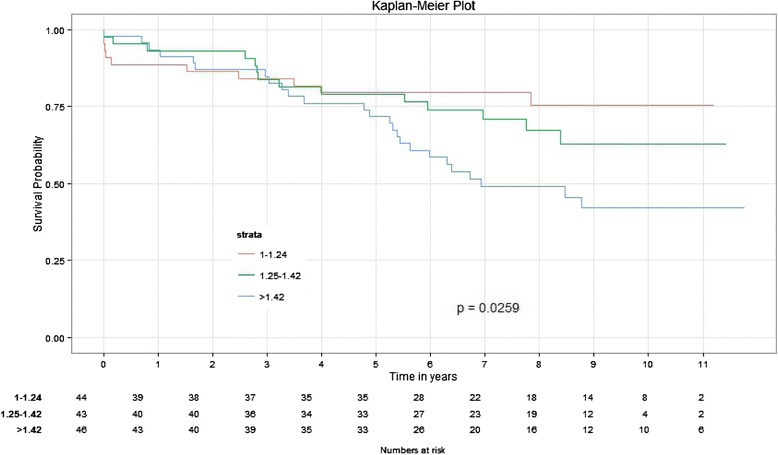
Survival probabilities between Scar groups based on LVSS (LVSS 1–1.24, LVSS 1.25–1.42 and LVSS >1.42)

Number of segments of scar (*p* = 0.005, HR = 1.16 CI = 1.05–1.28), Number of Trans mural segments (*p* = 0.001, HR = 1.2 CI = 1.07–1.33) and LVSS (*p* = 0.0008, HR = 4.9 CI = 1.9–12.3) were independently associated with long-term mortality. Groups based on LVSS (LVSS 1–1.24 (*n* = 44), LVSS 1.25–1.42 (*n* = 43), and LVSS >1.42 (*n* = 46)) had significantly different survival probabilities (Fig. 4) during the follow up (*p* = 0.026), favoring smaller LVSS.

## Discussion

To the best of our knowledge this is the longest survival follow up after CMR and CABG stratified based on scar burden. Data was retrievable in 94.4 % of our study population accurately. The study population had a long-term mortality of 35 % over a period of 8.3 years. In prior studies including patients with ischemic cardiomyopathy (EF 26–29 %) and CABG, mortality ranged from 29–58 % over 10 years [19, 20]. In previous studies, patients with ischemic HF who had CMR, mortality was 15 % over a follow up of 2.6 years [10] and 41 % over a follow up of 5.8 years [23]. Our patient group had a moderately reduced LVEF (median 38 %), an increased prevalence of prior MI, and a longer follow up with comparable mortality to prior studies.

In our study, there was no significant difference in survival based on presence or absence of scar. The most common reason for ordering CMR prior to formulating treatment strategy in patients with angiographically significant CAD is for assessment of scar/viability. Myocardial viability however, is a complex and incompletely understood phenomenon. In clinical practice, the presence of visually significant CAD on angiography in patients with cardiomyopathy is equated as ischemic cardiomyopathy. Previous studies on patients with ischemic versus non-ischemic cardiomyopathies have shown that the extent of CAD is the most predictive feature of prognosis [24]. However, this may not be the case in all patients, and in a subgroup of patients with obstructive CAD, there may be a concomitant non-ischemic etiology for cardiomyopathy. CMR may be useful in identifying this subgroup. In our study, patients with no scar had significantly lower prevalence of prior MI as well as a lower median number of totally occluded major epicardial arteries compared to the scar group. While no scar on CMR may indicate that all segments are viable in these patients, it may alternately suggest a non-ischemic cardiomyopathy as the primary etiology. This may suggest the need for further analysis to prove ischemia in this group of patients undergoing CABG, and further study of non-scar patients to evaluate them for intrinsic myocardial abnormalities. The current study is limited by ischemia data acquired before the clinical decision for CABG was made and the control arm to identify the subgroup of patients with in the no scar group that may have benefited from revascularization.

In the scar group patients, a higher scar burden is negatively associated with survival despite CABG. Several factors could explain this. Prior studies suggested that there could be a scar threshold beyond which the benefits of revascularization are attenuated. It is possible that patients with large amount of scar in the revascularized territory may actually not have significant viable tissue that can be recovered. In addition, previous studies have shown that myocardial scar size and characteristics predict spontaneous ventricular arrhythmias in patients with an ischemic cardiomyopathy [25–28] which can result in worse survival independent of LV functional improvement. In this study we have used several easily reproducible parameters to assess scar burden. Number of segments of scar and number of segments with significant transmurality of scar are easy to measure and are independently associated with long term mortality, but they may have potential for underestimating and sometimes overestimating global scar burden. To overcome this we have used LV scar score, another parameter which is easy to measure and a better index of global scar burden. LVSS also is independently associated with survival. While the scar group was subdivided based on study sample size and previously available data, it is somewhat arbitrary. However, the overall study results in the entire scar group strongly suggest a worse prognosis with increased scar burden. The favorable prognosis of patients with small scar burden likely represents patients with proven ischemic CM who then obtain the full benefit of revascularization.

### Clinical implications

Multiple prior studies suggest worse outcomes and survival in patients with LGE on CMR. There is data to support functional improvement after CABG [15–18]. CABG aims to improve symptoms, LVEF, MACE and prolong survival by reducing ischemia. Data from observational studies suggest significant survival advantage from CABG over medical therapy in this patient population. The STICH (Surgical Treatment For Ischemic heart failure) study failed to show improvement in all-cause mortality but there was a decrease in cardiovascular death with CABG [21]. LGE on CMR provides unique long-term prognostic information after CABG, independent of traditional predictors. Randomized studies comparing therapeutic alternatives using CMR viability data would be needed to determine the utility of this information in deciding on treatment strategy. The study used simplified quantification tools to calculate fibrosis burden based on prior published data, to allow a quick and day to day estimation of LGE and prognostication in clinical medicine.

### Study limitations

There are several limitations for this study. This study is a retrospective observational design at a single center. As patients were referred by treating physicians, there could be potential for referral bias in a tertiary care setting and thus the results may not be generalized to all patients undergoing CABG. Patients, in whom the treating physician favored medical therapy after reviewing the CMR results, likely those with large scar area, would not have been evaluated in this study leading to a selection bias. The data on ischemia is not available in these patients which could have added more clarity to the outcomes in different subgroups. The currently ongoing Ischemia study may provide more understanding in this regard. Change of contrast agent during the study period could potentially effect the LGE quantification; however, to the best of our knowledge this effect is minimal and is uniform between different subgroups in the study. Cause of death was not identified in our study. While it is possible that deaths could have occurred from non-cardiovascular causes, we believe that it is more meaningful to study all-cause mortality for patient outcomes. In addition, retrospective identification of cause of death can be highly inaccurate and can adversely affect interpretation of results [29]. Data was not available in 5.6 % of the study group patients and is unlikely to alter the results of the study.

## Conclusions

In patients undergoing CABG for significant CAD and have myocardial scar by CMR, the amount of scar is negatively associated with survival, independent of traditional predictors. Patients with significant CAD but without CMR evidence of MI had no statistically significant difference in survival after CABG compared to those with scar.

## Abbreviations

ACE, angiotensin convertase enzyme; BMI, body mass index; CABG, coronary artery bypass graft surgery; CAD, coronary artery disease; CMR, cardiovascular magnetic resonance; ESVi, end systolic volume index; HF, heart failure; LGE, late gadolinium enhancement; LV, left ventricular; LVEDV, left ventricular end diastolic volume; LVEF, left ventricular ejection fraction; LVEF, left ventricular ejection fraction; LVESV, Left ventricular end systolic volume; LVSS, left ventricular scar score; MACE, major adverse cardiac events; MI, myocardial infarction; MVO, micro vascular obstruction; NICM, non‐ischemic cardiomyopathy; PAD, peripheral artery disease; STICH surgical treatment for ischemic heart failure; STS, society of thoracic surgery
